# Does environmental adaptation or dispersal history explain the geographical distribution of *Ixodes ricinus* and *Ixodes persulcatus* ticks in Finland?

**DOI:** 10.1002/ece3.9538

**Published:** 2022-12-12

**Authors:** Niko Kulha, Kalle Ruokolainen, Eero J. Vesterinen, Maija Lamppu, Tero Klemola, Jani J. Sormunen

**Affiliations:** ^1^ Biodiversity Unit Zoological Museum University of Turku Turku Finland; ^2^ Natural Resources Institute Finland (Luke) Helsinki Finland; ^3^ Department of Biology University of Turku Turku Finland; ^4^ Institute of Biology University of Neuchâtel Neuchâtel Switzerland

**Keywords:** dispersal history, environmental adaptation, Finland, geographical distribution, *Ixodes persulcatus*, *Ixodes ricinus*, spatial autocorrelation, spatial randomization

## Abstract

In Finland, the distribution area of the taiga tick, *Ixodes persulcatus* (Schulze, 1930), is nested within a broader area of distribution of a congeneric species, the sheep tick, *Ixodes ricinus* (Linnaeus, 1758) (Acari: Ixodidae). We assess whether distinct environmental adaptations or dispersal history provides a more parsimonious explanation for the differences in the distributions of the two common and medically important ixodids in Finland. We used an innovative spatially constrained randomization procedure to analyze whether crowdsourced occurrence data points of the two tick species had statistically different associations with any of the 28 environmental variables. Using points of presence in a region of species co‐occurrence, we built Maxent models to examine whether environmental factors or dispersal history could explain the absence of *I. persulcatus* in a part of the range of *I. ricinus* in Finland. Five environmental variables—number of inhabitants, road length, elevation above sea level, proportion of barren bedrock and boulders, and proportion of unsorted glacial deposits—were significant at *p* ≤ .05, indicating greater between‐species difference in original than in the randomized data. Of these variables, only the optimum value for unsorted glacial deposits was higher for *I. persulcatus* than for *I. ricinus*. Maxent models also predicted high relative habitat suitability (suitability >80%) for *I. persulcatus* south of its current, sharply bounded distribution range, suggesting that the species has not fulfilled its distribution potential in Finland. The two most common and medically relevant ixodids in Finland may colonize habitats with different environmental conditions. On the contrary, the recent establishment and ongoing dispersion of *I. persulcatus* in Fennoscandia rather than environmental conditions cause the southernmost distribution limit of the species in Finland.

## Graphical abstract

We examined whether distinct environmental adaptations or dispersal history provides a more parsimonious explanation for the differences in the distributions of Ixodes ricinus and Ixodes persulcatus in Finland. We found evidence that the two most common and medically relevant ixodes ticks in Finland may colonize habitats with different environmental conditions. However, recent establishment and ongoing dispersion of I. persulcatus in Fennoscandia rather than environmental conditions causes the southernmost distribution limit of the species in Finland.

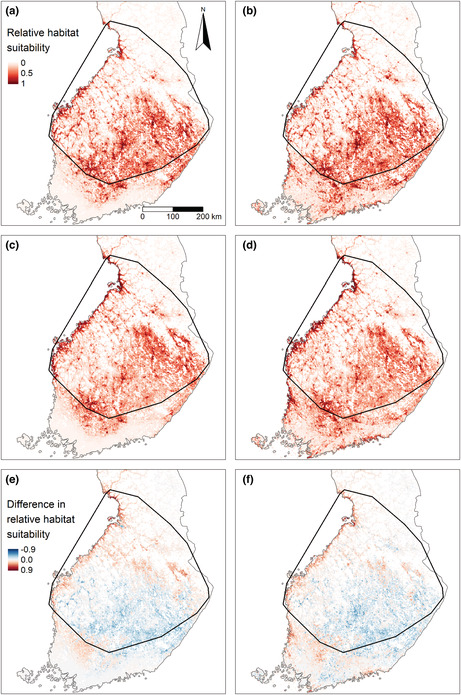

## 1 INTRODUCTION

Tick‐borne diseases mediated by hard ticks (Acari: Ixodidae) constitute a growing threat to public health in Europe (Marques et al., 2021). Ticks serve as vectors for a variety of pathogens (Capligina et al., 2020; Katargina et al., 2015; Sormunen, Kulha, et al., 2020), of which the *Borrelia burgdorferi* sensu lato bacteria group (causative agents for Lyme borreliosis) and the tick‐borne encephalitis virus are the most serious from a human health perspective (Bogovic & Strle, 2015). In northwestern Eurasia, the main vectors for these pathogens are two tick species: the sheep tick, *Ixodes ricinus* (Linnaeus, 1758), and the taiga tick, *Ixodes persulcatus* (Schulze, 1930) (Laaksonen et al., 2018; Marques et al., 2021).

*Ixodes ricinus* and *I. persulcatus* have been shown to co‐occur in western Russia (Tokarevich et al., 2011) and in five European nations west from Russia: Estonia (Katargina et al., 2015), Finland (Laaksonen et al., 2017; Sormunen, Andersson, et al., 2020), Hungary (Hornok et al., 2020), Latvia (Capligina et al., 2020), and Sweden (Jaenson et al., 2016). Increase in abundance and expansion of the range of distribution have been reported for both species especially in the mentioned Fennoscandian regions (Finland, Sweden, and western Russia) during the past few decades (Bugmyrin et al., 2012; Tokarevich et al., 2011). Particularly the northwestern expansion of *I. persulcatus* that originates from western Russia appears relatively recent and rapid (Tokarevich et al., 2011), with the first established Swedish population reported from the northeastern parts of the country in 2015 (Jaenson et al., 2016). In Finland, *I. persulcatus* appears to have established during the late 20th century, although the lack of historical data on the occurrence of the species hampers assessing the exact timing of establishment (Öhman, 1961). For *I. ricinus*, historical records suggest that the species has occurred in Finland for centuries (Hulden, 2007) even if the distribution of the species was demonstrated with taxonomically and spatially reliable documentation only in the 1950s (Öhman, 1961).

**FIGURE 1:**
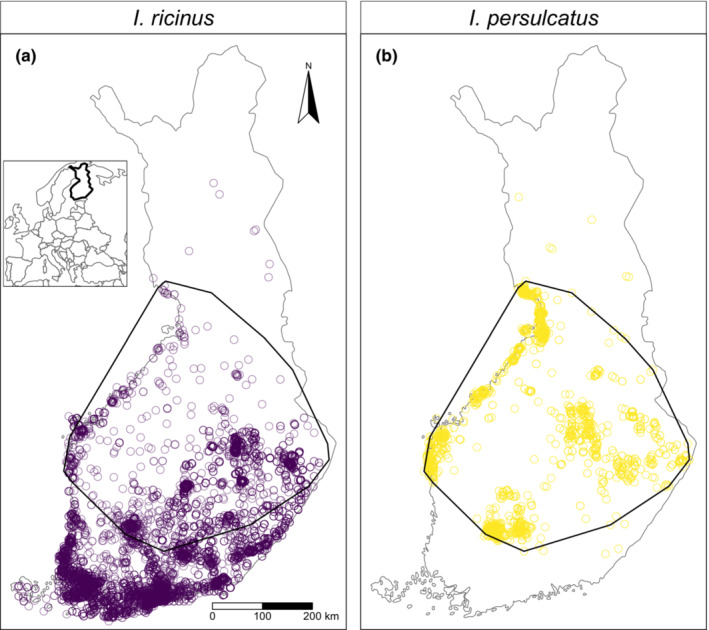
The occurrence points of *Ixodes ricinus* (a) and *Ixodes persulcatus* (b) in the crowdsourced data. The black polygon indicates the area of co‐occurrence for the two species. Only the observations within the area of co‐occurrence were used in this study.

The modern geographical distribution of *I. persulcatus* and *I. ricinus* in Finland has been clarified with the help of a nationwide crowdsourcing campaign (Laaksonen et al., 2017). It was found that the area of distribution of *I. persulcatus* is nested within the distribution area of *I. ricinus* (Figure 1a). Also, the southern distribution limit of *I. persulcatus* was remarkably sharp and some of the patches where the species showed the highest density of occurrence lay at this distribution boundary (Figure 1b). The distribution boundary does not follow any prominent landform feature. Instead, the most obvious environmental explanation for the placement of the distribution boundary would be climate because the hemiboreal climate of southwestern Finland gradually turns to boreal climate as one advances toward north and northeast. In Eurasia, *I. persulcatus* has a more continental distribution than *I. ricinus* (Kovalev et al., 2015; Livanova et al., 2016). Therefore, it seems plausible that it is better adapted to dry and cold climates compared with *I. ricinus* (Korenberg et al., 2021). However, this climatic hypothesis is hardly the sole explanation for the southernmost distribution limit of *I. persulcatus* in Finland, as the species occurs and even dominates in certain regions of neighboring Estonia (Katargina et al., 2015) and Latvia (Capligina et al., 2020) where temperature sums, vegetation periods and amounts of precipitation are comparable to those in southern Finland.

In terms of biogeographical theory, our research question is an example of weighing among three alternative but not mutually exclusive explanations for spatial patterns of species distribution. Firstly, according to the niche theory (e.g., Grinnell, 1917; Hutchinson, 1957; MacArthur, 1972), every species may maintain a persistent population only in specific environmental conditions. Consequently, a viable population of a given species occurs only in such geographical areas where these environmental conditions—physical, chemical, and biological—are met (Chase & Leibold, 2003). Secondly, a dispersal barrier may prevent a species from occupying locations that are environmentally suitable for it. In our case, no obvious barrier, such as a mountain range or a large water body, exists. Instead, more subtle environmental factors related to, e.g., climate, soil, or vegetation structure may act as a less obvious dispersal barrier. If a barrier is removed (e.g., due to climate change) or the species manages to surpass it, the range of the species would—at least for a while—show a front that advances to fill the suitable habitat that had become available for the species. Thirdly, the so‐called neutral theory of biogeography (henceforth neutral theory) can be regarded as a null hypothesis for explaining patterns of species distribution (Hubbell, 2001). The neutral theory states that the observed distribution of a given species results from an ongoing process of random but spatially constrained dispersal of propagules followed by a success of establishment and reproduction that is independent of the quality of the environment.

The central question in our study is whether the absence of *I. persulcatus* in southern Finland is due to a difference in environmental requirements between the two tick species or whether it only shows the position of a dispersal front that eventually will advance southwards. Statistically reliable evaluation of the possible differences in environmental requirements between *I. persulcatus* and *I. ricinus* requires that the spatial autocorrelation in their occurrence data is explicitly considered (Keitt et al., 2002; Legendre, 1993). A common approach for dealing with inflated type‐I error rates due to spatial autocorrelation is to perform a restricted randomization test that preserves the spatial structure of the data. In datasets that make a regular geographical grid, this can be achieved with toroidal shift methods (Harms et al., 2001). When the sampled data are irregularly spaced, as it is in our case, the most elegant way would be the Moran spectral randomization (MRS) that generates random variables that retain the spatial structure of the original samples (Wagner & Dray, 2015). In our tick data, the MRS approach was not applicable because it cannot handle a situation in which the occurrences of two species have to be randomized simultaneously, keeping the number of co‐occurrences constant. For this reason, we developed and implemented a novel spatially constrained randomization procedure to examine the environmental requirements and range of distribution of the two ixodids.

Starting from the premises laid by biogeographical theories for spatial patterns of species distribution, we examined the role of environmental factors and distribution history in explaining the distribution patterns of *I. ricinus* and *I. persulcatus* in Finland. Specifically, we asked whether: (1) environmental conditions are different in localities where the two species were observed in the region of species co‐occurrence, and (2) whether environmental factors or dispersal history explain the absence of *I. persulcatus* from southern Finland.

## 2 MATERIALS AND METHODS

### 2.1 Tick data

We used material from the crowdsourcing campaign coordinated by the University of Turku Tick Project to examine the factors influencing the distribution of *I. ricinus* and *I. persulcatus* in Finland. In the campaign, ordinary citizens were asked to mail ticks they encountered to the University of Turku for pathogen analysis. The observers were also asked to provide geographical coordinates of the sites where ticks were collected. The campaign resulted in approx. 20,000 individual ticks sent to the university in approx. 7000 shipments between May and September 2015 (Laaksonen et al., 2017, 2018). Of the ca. 20,000 ticks, 17,936 were morphologically identified to species (14,133 *I. ricinus*, 3803 *I. persulcatus*). A screening of approx. 3800 of the samples with duplex qPCR assay identified 35 *I. ricinus* × *I. persulcatus* hybrids (Laaksonen et al., 2018). Adults (95.5%) were the most sampled stage of tick development, followed by nymphs and larvae (4.4% and 0.1%, respectively; Laaksonen et al., 2017).

We removed individuals lacking spatial information and converted the remaining observations to presence data by removing eventual multiple individuals of the same species in one locality. In order to answer our research questions, we geographically pruned the data to the area of species co‐occurrence. We delineated this area by defining a minimum bounding geometry around the locations from where individuals of *I. persulcatus* were recorded. Six geographically disparate tick observations were recorded north and four south from the main bulk of *I. persulcatus* presence. These observations were treated as spatial outliers and were not considered in defining the minimum bounding geometry. We removed observations of *I. ricinus* outside the area of species co‐occurrence. After pruning, our data consisted of 1232 occurrence points of *I. ricinus* (Figure 1a) and 1078 of *I. persulcatus* (Figure 1b) from the area of species co‐occurrence. None of the occurrence points contained observations of both species. We regard this a relevant feature of the data as there were many records of more than one individual, and therefore potentially two species, observed in a given geographical location.

### 2.2 Environmental variables

Numerous environmental factors regulate the distribution of *I. ricinus* and *I. persulcatus* by influencing their host‐seeking possibilities and mortality rates (Medlock et al., 2013; Sirotkin & Korenberg, 2018). Here, we used a set of 28 environmental variables that have been shown to influence the occurrence and survival of the two ixodids. Moreover, we included variables describing human population density and the number of summer cottages that might influence the probability of observation in crowdsourced data (Welvaert & Caley, 2016). The explanatory variables describe soils, surface water cover, land use, climate, vegetation, human presence, and topography. A list of the explanatory data and data sources is found in Appendix.

We obtained an environmental characterization for each tick occurrence point by drawing a circular buffer with the radius of 1 km around each point and calculating statistics for each used environmental variable within the buffer (Table A1). We used the buffers to diminish the effect of probable inaccuracy in the geographical coordinates of the crowdsourced data.

### 2.3 Differences in habitat preferences between *I. ricinus* and *I. persulcatus*

Our null hypothesis is that between *I. ricinus* and *I. persulcatus* there is no difference in the optima along different environmental gradients. To test this hypothesis, we first calculated the observed difference in optimum between the species along each of the 28 environmental variables. Here, optimum was defined as the average of the values recorded for the occurrence points of the species. The spatial distributions of both *I. ricinus* and *I. persulcatus* were autocorrelated (Moran's *I* 0.02 and 0.01, *p* < .01 and < .01, respectively; tested with the package “ape,” Paradis & Schliep, 2019). Accordingly, statistically reliable evaluation of the differences in environmental requirements between *I. persulcatus* and *I. ricinus* necessitates that the spatial autocorrelation in their occurrence data is explicitly considered. Moran's spectral randomization (Wagner & Dray, 2015) is a powerful method for generating randomized data while maintaining its original spatial structure. However, this randomization procedure was not applicable here because at least currently it does not allow the generation of randomized spatial structure in multi‐species occurrence data that are constrained to maintain the spatial pattern of co‐occurrence among the species. In our data, the observed pattern was that the two *Ixodes* species were never found together in the same location.

To account for the spatial autocorrelation, we developed a novel spatially constrained randomization approach that produces randomized occurrence points for the two tick species in such a way that the random points reproduce as precisely as possible the spatial pattern that we observed for *I. ricinus* and *I. persulcatus* simultaneously. A Euclidean distance matrix describes the spatial pattern of the occurrence points and, consequently, our task was to generate randomly chosen geographical coordinates of the 1232 and 1078 occurrence points so as to produce two Euclidean distance matrices that contain similar selections of distance values as possible to the two original distance matrices.

**FIGURE A1:**
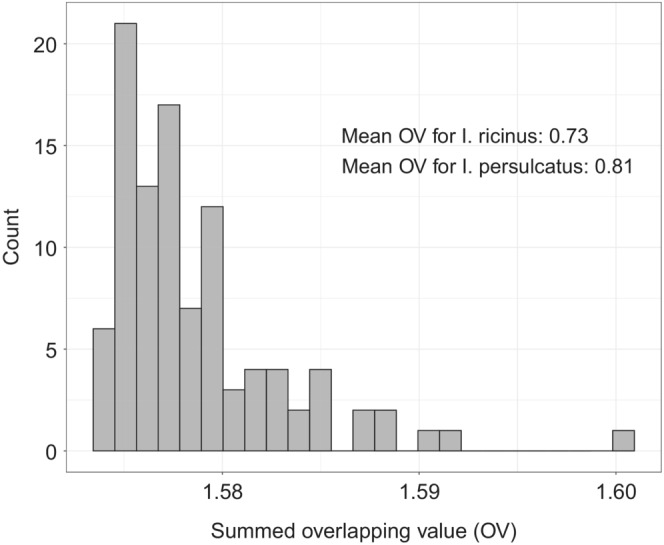
Histogram of the summed overlapping values for the 100 draws with the highest summed overlapping values and the species‐specific means of the overlap values in the 100 draws.

To produce such distance matrices, we randomized the tick occurrence points 10,000 times, each time separating them into two sets with 1232 and 1078 points, corresponding to *N* of *I. ricinus* and *I. persulcatus*, respectively. We calculated Euclidean distance matrices based on each of the randomized sets of occurrence points (henceforth randomized distance matrices). We quantified the similarity of each randomized distance matrix to its corresponding original distance matrix by calculating an overlapping index *η* between the matrices (Pastore, 2018). The overlapping index is a distribution‐insensitive measure of similarity between two distributions, where the similarity of distributions is defined as the proportion of overlapping area between two distributions (Pastore & Calcagni, 2019). The value of *η* varies from zero (no overlap) to one (complete overlap). After each random draw, we summed the *η* calculated separately for *I. ricinus* and *I. persulcatus* and retained the 100 draws with highest summed *η* for further analysis (Figure A1). We used the package “overlapping” (Pastore, 2018) to calculate the overlapping index.

We estimated the probability of type‐I error (*p*) in rejecting the null hypothesis of no difference in the optimum along an environmental gradient between *I. ricinus* and *I. persulcatus* as follows. We calculated the arithmetic mean of each environmental variable in the sites of observation of either species, subtracted the means, and quantified the absolute values of these subtractions. This calculation resulted in an absolute difference of means of a specific environmental variable in sites where *I. ricinus* or *I. persulcatus* were observed. Next, we performed the same calculation for each of the 100 random draws with highest summed *η*. For each environmental variable, we recorded the number of random draws where the absolute value of subtraction was larger than or equal to that of the original data. This number divided by the total number of draws plus one (representing the original observation) gave us the estimated *p*.

In the hypothetical case that our 28 environmental variables were mutually uncorrelated, we would by chance expect to observe 1.4 variables with a between‐species difference at a type‐I risk level of 0.05 (28 × 0.05 = 1.4). Naturally, our explanatory variables are not mutually independent. To define the effective number of uncorrelated environmental dimensions, we performed a principal component analysis (PCA) on all explanatory variables used in this study, after standardizing each variable's average to zero and variance to one. Similarly, we performed a PCA with variables for which our estimated *p* was below 5% so that we could estimate their effective number and see whether it was greater than one would expect to observe by chance only. We used the package “factoextra” for PCA (Kassambra & Mundt, 2020).

### 2.4 Absence of *I. persulcatus* from southern Finland

To explore whether environmental factors used in this study explain the absence of *I. persulcatus* from southern Finland (the second research question), we created niche models for both *I. ricinus* and *I. persulcatus* using data from the area of species co‐occurrence and predicted the probability of species occurrence in the whole of Finland. Due to the limited occurrence of either species north of the 66th parallel north (Hvidsten et al., 2014; Laaksonen et al., 2017), we used the location of the center point of Rovaniemi football stadium (approximately N° 66) as the northernmost prediction limit. The aim of this niche modeling was to explore whether the model for *I. persulcatus* in the area south from its distribution limit predicts a lower probability of occurrence than the model for *I. ricinus* in the same area. Such a difference in the model predictions would suggest that the absence of *I. persulcatus* in southern Finland is due to one or more of the environmental factors included in this study, whereas a lack of difference in models would suggest dispersal limitation, or an environmental factor not included in this study.

We used the maximum entropy method (Maxent) in package “dismo” (Hijmans et al., 2021) to create the niche models. Maxent is a general‐purpose machine learning method, which applies the maximum entropy principle (Jaynes, 1957) for fitting a niche model in which the estimated species distribution is as close to a uniform distribution as it is possible while still being able to explain the observed dependences between occurrence points and environmental variables. Maxent is designed to be used with presence‐only species observations and a set of environmental variables from the region of interest (Philips et al., 2006). Maxent predictions provide estimates of relative habitat suitability, not estimates of occupancy probability (Guillera‐Arroita et al., 2014).

We fitted two kinds of models for both species: (1) a model with each environmental variable used in this study as a covariate (henceforth full model), and (2) a model excluding climatic variables (growing season length, temperature sum of growing season, precipitation sum of growing season, mean temperature, and snow season length, henceforth pruned model). By leaving out the climatic variables from the pruned model we aimed at avoiding climatic extrapolation in the area south of the distribution boundary of *I. persulcatus*, where several climatic features are known to be different than elsewhere in Finland. To match the grain of the environmental variables to that of the data for species observations (a circular buffer with 1 km radius, area ≈ 3.14 km^2^), we created a grid where the cell size (1.772 km × 1.772 km) matched the buffer area. Similar to the buffers, we extracted the environmental variables to grid cells and rasterized the grids to be used in models. During each model fit, we retained a random sample of 20% of observations for model validation. We used the regularization value of 1 in each model fit. We validated the models using area under the curve value (AUC) generated using the validation data (Liu et al., 2011). The AUC values of 0.6–0.7 indicate a fair, 0.7–0.8 good, 0.8–0.9 very good, and > 0.9 excellent model classification accuracy (Swets, 1988).

We performed all data manipulation and calculations in R 4.0.4 (R Core Team, 2021). We used the package “tmap” for drawing maps (Tennekes, 2018), “ggplot2” (Wickham, 2016) for drawing other figures, and packages “sf” (Pebesma, 2018) and “raster” (Hijmans, 2022) for various processing and calculation of spatial information in vector and raster formats, respectively.

## 3 RESULTS

### 3.1 Differences in habitat preferences between *I. ricinus* and *I. persulcatus*

**FIGURE 2:**
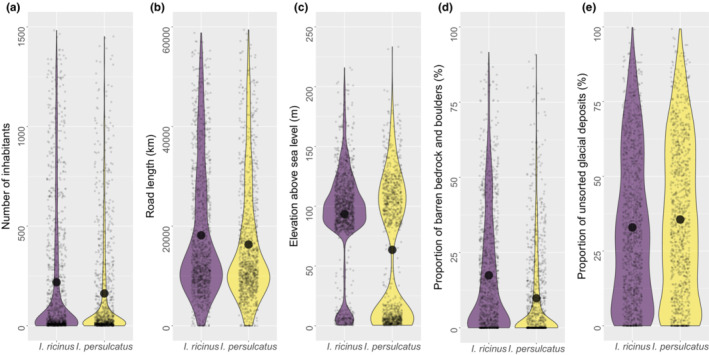
The distributions of environmental variables with estimated *p* < 5% for both species: (a) population, (b) summed road length, (c) elevation, (d) rockiness, and (e) moraine.

**FIGURE A2:**
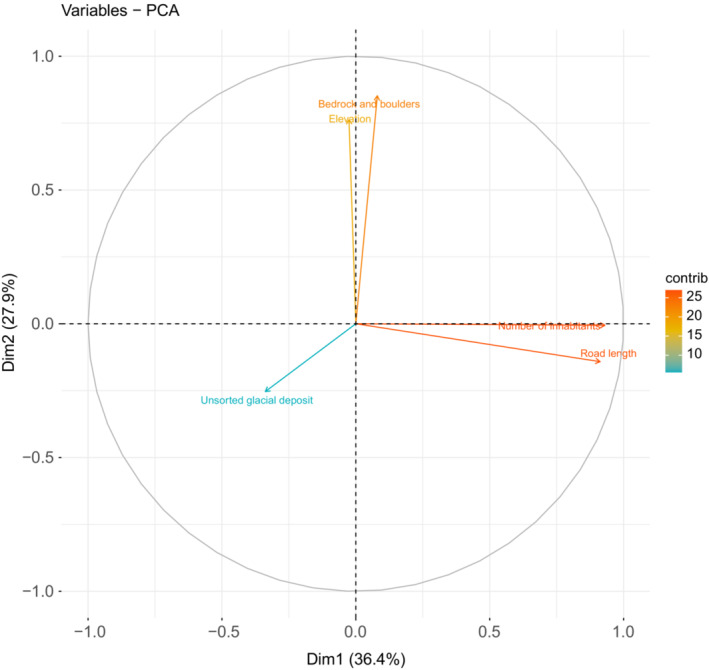
Principal component analysis of the five variables with the estimated *p* < 5%.

Out of the examined 28 environmental variables, four had an estimated *p* < 1% and one <5% (Figure 2, Table 1). These variables were the number of inhabitants, road length, elevation above sea level (henceforth elevation), proportion of barren bedrock and boulders (henceforth rockiness), and proportion of unsorted glacial deposits (henceforth moraine). These five variables were partially intercorrelated and a conservative estimate at 5% level of risk is that together they effectively represent between two and three independent dimensions of environmental variation (Table A2, Figure A2). This is more than one would expect to observe by chance only. A principal component analysis of the 28 environmental variables showed that effectively the environmental space can be represented by 18 independent dimensions (>95% of the total variation is captured by the first 18 principal components, Table A3). Hence, one would expect to observe an environmental difference between the two tick species along approx. One independent environmental dimension at the *p* ≤ .05 level (18 × 0.05 = 0.9).

**TABLE 1:** Differences in environmental associations between *Ixodes ricinus* and *Ixodes persulcatus* in are of species co‐occurrence. Estimated *p* < 5% indicates a significant difference between species.

Variable	Estimated *p* (%)	Species with greater optimum value
Number of inhabitants	0.99	*I. ricinus*
Road length	0.99	*I. ricinus*
Elevation	1.98	*I. ricinus*
Bedrock and boulders	0.99	*I. ricinus*
Unsorted glacial deposits	0.99	*I. persulcatus*
Coarse‐grained soil	25.74	*I. persulcatus*
Fine‐grained soil	76.24	*I. ricinus*
Sludge	74.26	*I. persulcatus*
Clay	26.73	*I. persulcatus*
Organic soil	26.73	*I. persulcatus*
Lake	26.73	*I. ricinus*
Sea	70.30	*I. persulcatus*
Surface water	28.71	*I. persulcatus*
River	26.73	*I. persulcatus*
Growing season length	62.38	*I. ricinus*
Temperature sum of growing season	26.73	*I. ricinus*
Precipitation sum of growing season	26.73	*I. ricinus*
Mean temperature	23.32	*I. ricinus*
Snow season length	28.71	*I. ricinus*
Tree canopy cover	18.81	*I. ricinus*
Proportion of deciduous trees	32.67	*I. ricinus*
Mean tree age	26.73	*I. persulcatus*
SD of tree age	39.60	*I. persulcatus*
Proportion of young forest	99.01	*I. persulcatus*
Summer cottages	99.01	*I. persulcatus*
Urban area	26.73	*I. ricinus*
Arable land	28.71	*I. persulcatus*
Peatland	97.03	*I. persulcatus*

**FIGURE A3:**
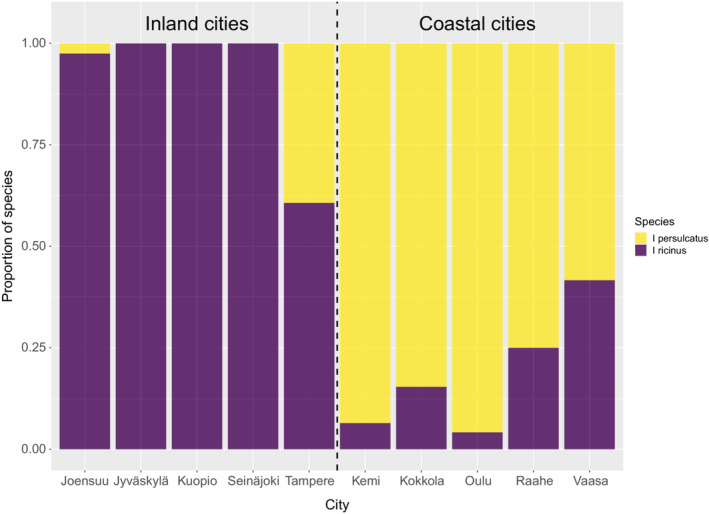
Proportions of *I. ricinus* and *I. persulcatus* in five inland and coastal cities with highest number of inhabitants located in the area of species co‐occurrence. According to a chi‐square test of independence, the difference in the proportion of *I. ricinus* differed significantly between the coastal and inland cities (χ^2^ = 11.3, *df* = 4, *p* = .023).

Of the five environmental variables with *p* < .05, four showed a higher optimum value for *I. ricinus* than for *I. persulcatus* (Figure 2, Table 1). The number of inhabitants and road length can be regarded as proxies for anthropogenic factors, and they were generally higher in areas where *I. ricinus* were observed. However, *I. persulcatus* was the dominant species in major coastal cities, whereas *I. ricinus* prevailed in major inland cities (chi‐square test of independence χ^2^ = 11.3, *df* = 4, *p* = .023) (Figure A3).

### 3.2 Absence of *I. persulcatus* from southern Finland

**FIGURE 3:**
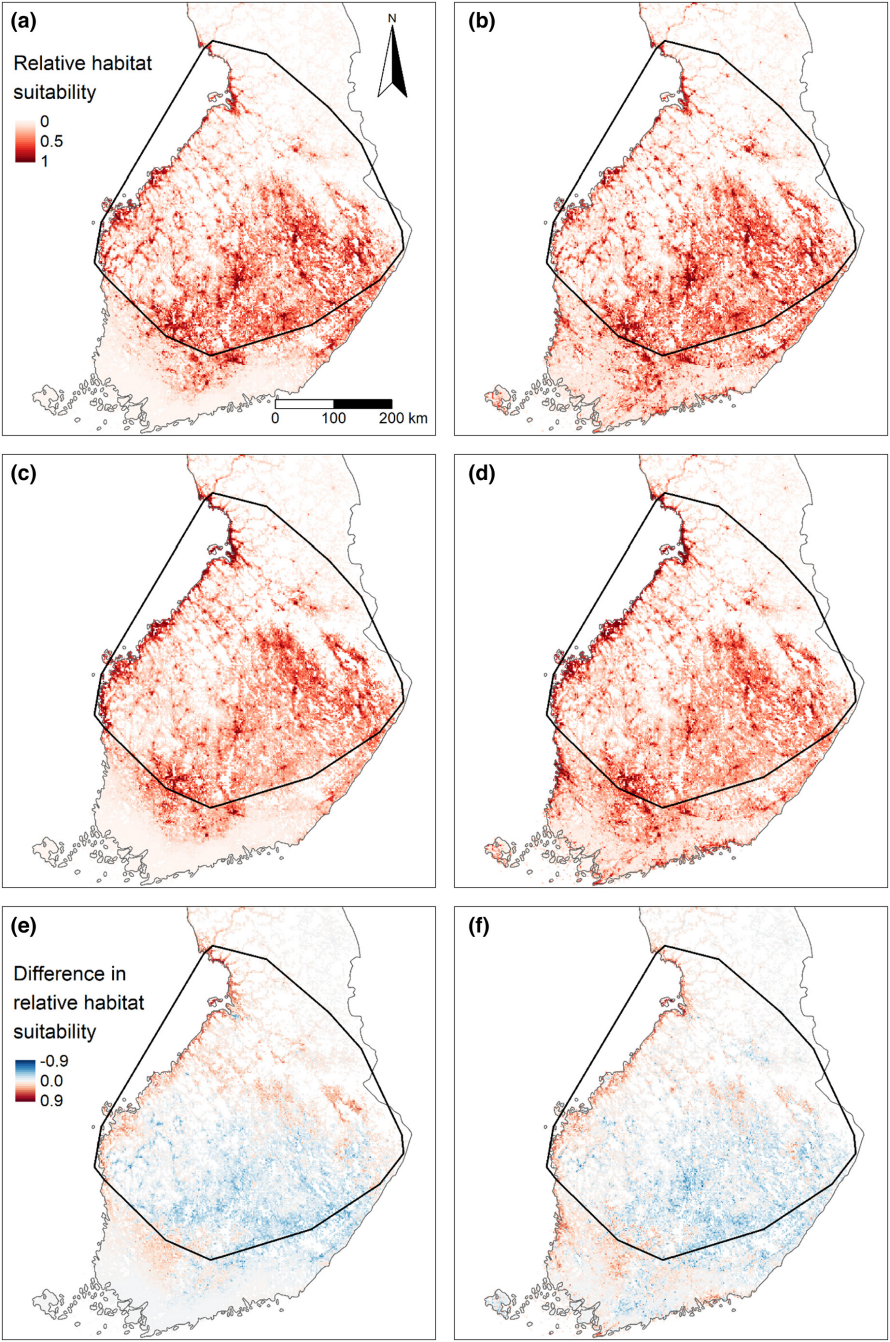
Predictions of relative habitat suitability for *Ixodes ricinus* (a–b) and *Ixodes persulcatus* (c–d), derived from full (a, c) and pruned (b, d) models. Maps e–f show the difference in relative habitat suitability between the species, based on predictions from full (e) and pruned (f) models. We calculated the difference in relative habitat suitability by subtracting the full and pruned model predictions for *I. ricinus* from the corresponding predictions for *I. persulcatus*. Hence, in maps e–f, red indicates areas with higher relative habitat suitability for *I. persulcatus* and blue for *I. ricinus*.

All maximum entropy model predictions indicated similar patterns of relative habitat suitability for *I. ricinus* (Figure 3a,b) and *I. persulcatus* (Figure 3c,d). In predictions, relative habitat suitability for *I. persulcatus* was high (>0.80) also south of its current southernmost distribution limit in Finland, especially when the climate variables were excluded from the model (Figure 3d). The predictions from full models indicated low relative habitat suitability for both ixodids in lower latitudinal parts of Finland (Figure 3a,c). In both models, relative habitat suitability was higher for *I. persulcatus* than for *I. ricinus* in the coastal regions, whereas relative habitat suitability was higher for *I. ricinus* than for *I. persulcatus* in most inland regions (Figure 3e,f).

The proportion of urban area had the highest contribution in all Maxent models, despite model type and modeled species (Table 2). The number of inhabitants was also among the predictors with the highest contribution in all models. The other variables with highest contributions varied depending on the model type and modeled species.

**TABLE 2:** The five variables with highest variable contribution (%) for both full and pruned models for both species.

Model type			
Full model		Pruned model	
*Ixodes ricinus*	*Ixodes persulcatus*	*I. ricinus*	*I. persulcatus*
Urban area 20.1	Urban area 20.7	Urban area 27.9	Urban area 26.1
Organic soil 18.7	Elevation 20.2	Number of inhabitants 15.6	Elevation 23.4
Number of inhabitants 12.7	Growing season length 17.1	Organic soil 10.6	Number of inhabitants 12.2
Growing season length 17.0	Growing season temperature sum 11.7	Lake 9.2	Tree canopy cover 6.5
Growing season temperature sum 12.7	Number of inhabitants 5.1	Proportion of deciduous trees 8.0	Proportion of deciduous trees 5.7

For the full models, the AUC calculated with validation data was 0.829 for *I. ricinus* and 0.844 for *I. persulcatus*. The AUC of pruned models was 0.813 for *I. ricinus* and 0.830 for *I. persulcatus*.

## 4 DISCUSSION

### 4.1 Differences in habitat preferences between *I. ricinus* and *I. persulcatus*

We observed differences in environmental conditions in localities where *I. ricinus* and *I. persulcatus* were present within the area of species co‐occurrence. The sites where *I. ricinus* was present had higher rockiness and elevation than the sites in which *I. persulcatus* was observed. However, the sites with *I. persulcatus* had a higher proportion of moraine than sites where *I. ricinus* occurred. In Fennoscandia, sites with a high proportion of moraine typically have low topographic positions, contrary to rocky and elevated areas (Hirvas & Nenonen, 1985). Moreover, the rocky and elevated sites often have thin or nonexistent soils and are thus poorer and more xeric than sites with the high proportion of glacial tills (Roiko‐Jokela, 1980; Seibert et al., 2007). This suggests that in their area of co‐occurrence, *I. ricinus* may colonize less fertile xeric sites than *I. persulcatus*.

The number of inhabitants and road length were higher in areas where *I. ricinus* was present than in areas where *I. persulcatus* occurred. Urban green spaces are known to sustain host species and to contain suitable habitats for ticks (Rizzoli et al., 2014), and tick populations that may carry out their full life cycles and form breeding populations have been observed in Finnish cities (Klemola et al., 2019; Sormunen, Kulha, et al., 2020). In general, our observation further suggests that anthropogenic factors may promote the occurrence of *I. ricinus* (Bugmyrin et al., 2012; Cayol et al., 2018).

Taken together, our results indicate that in the area of species co‐occurrence, *I. ricinus* is more resistant to desiccating conditions than *I. persulcatus* as it inhabits sites that are more prone to drought. Rocky and elevated sites with thin or nonexistent soils are naturally more xeric and prone to drought than low‐lying sites with more fine‐grained soils (Hirvas & Nenonen, 1985; Roiko‐Jokela, 1980; Seibert et al., 2007). Due to the urban heat island effect (UHI), urban areas typically have higher temperature than the surrounding rural areas (Kim, 1992). Increased evaporation rate caused by UHI together with effective drainage decrease moisture availability in urban areas compared with surrounding rural areas (Hage, 1975).

Our observation that *I. ricinus* was more often present in sites that are susceptible to drought compared with *I. persulcatus* may also be related to differences in temporal activity patterns between the two species. In our study region, *I. ricinus* has activity peaks approximately in May and August (Sormunen, Kulha, et al., 2020). *I. persulcatus* has a single activity peak in spring/early summer, after which its activity greatly diminishes or ceases altogether (Laaksonen et al., 2017; Pakanen et al., 2020; Tokarevich et al., 2011). As the activity period of *I. persulcatus* only lasts 2–3 months, they annually face a 9–10 months period of dormancy and overwintering in undergrowth/litter. During this time, *I. persulcatus* must survive on energy reserves acquired from a previous blood meal. Hence, they need to conserve energy as much as possible. In xeric sites, ticks likely face additional stress due to heat and drought (Sirotkin & Korenberg, 2018). This stress may force them to search for more favorable microenvironments and/or actively uptake water, both at the expense of their energy reserves. While the same stressors influence *I. ricinus*, their continued activity in autumn may provide more chances to find blood meals and thus replenish energy reserves before winter dormancy, which is shorter than that of *I. persulcatus*. This mechanism may lead to higher mortality rates in populations of *I. persulcatus* compared with *I. ricinus* in xeric environments.

Although the number of inhabitants and road length were higher in sites where *I. ricinus* occurred compared with sites where *I. persulcatus* was present, *I. ricinus* was not the dominant species in all cities. Specifically, *I. persulcatus* dominated over *I. ricinus* in coastal cities. The dominance of *I. persulcatus* over *I. ricinus*, has also been shown independently in the coastal city of Oulu (Pakanen et al., 2020). We propose that the main reason as to why *I. persulcatus* thrives in Finnish coastal cities is due to a reverse UHI. The geographical location and spatial context of a city, such as topography and proximity of large waterbodies, may influence the warming caused by UHI (Hjort et al., 2016). Consequently, the UHI effect may reverse in high‐latitude coastal cities (Suomi & Käyhkö, 2012). Moreover, the intensity of UHI correlates positively with city size (Zhou et al., 2017). Of the studied cities located on the relatively humid coastal plain of the northern Baltic Sea, Oulu is the only one with a population >70,000 (approx. 207,300 inhabitants in 2020).

Most environmental variables analyzed here did not significantly differ between sites where either *I. ricinus* or *I. persulcatus* were observed. For example, winter conditions were similar in locations where the two species were observed, despite the reportedly better cold adaptation of *I. persulcatus* compared with *I. ricinus* (Korenberg et al., 2021). *I. ricinus* and *I. persulcatus* are closely related species with broad environmental niches (Sirotkin & Korenberg, 2018). While the environmental factors analyzed here may influence the distributions of the two species in general, it is worth noting that the grain size of the used variables is not necessarily optimal for determining niche differences between *I. ricinus* and *I. persulcatus*. Environmental data with broad spatial coverage typically aim at capturing general patterns in environmental characteristics. Hence, such data may be suboptimal for capturing fine‐scale environmental variability (Turner & Gardner, 2015). However, variability in, e.g., microclimatic conditions is meaningful to off‐host ticks that respond to environmental differences also at very fine spatial scales (Van Gestel et al., 2022). Hence, it is possible that the occurrence of the two species is related to the environmental variables we analyzed, but the relationship was shrouded by too coarse data resolution.

### 4.2 Absence of *I. persulcatus* from southern Finland

The Maxent models gave very similar predictions for the geographical distribution of the two species. This modeled similarity is particularly relevant in the areas where *I. persulcatus* was absent in our data (i.e., southern Finland). Also, the Maxent models did not indicate the existence of any belt of unsuitable habitat at the southernmost distribution limit of *I. persulcatus*, which could act as a dispersal barrier for the species. These observations suggest that the current southernmost distribution limit of *I. persulcatus* in Finland is due to the distribution history of the species rather than the analyzed environmental factors. Naturally, we have not analyzed all environmental variables that potentially affect the distribution of Ixodes ticks and thereby explain the lack of *I. persulcatus* in southern Finland. Despite the full model predicting low relative habitat suitability for *I. persulcatus* in southern Finland, the absence of the species from the region is difficult to explain by climatic conditions, as the species occurs in neighboring Estonia (Katargina et al., 2015) and Latvia (Capligina et al., 2020) where temperature sums, amounts of precipitation, and the lengths of vegetation periods are similar to southern Finland.

The data analyzed here show a sharp southernmost distribution limit for *I. persulcatus* in Finland. While *I. ricinus* has occurred in Fennoscandia for decades to centuries (Hulden, 2007; Öhman, 1961), *I. persulcatus* appears to have established itself relatively recently in the region (Bugmyrin et al., 2012; Jaenson et al., 2016; Laaksonen et al., 2017). Western (Tokarevich et al., 2011) and central Russia (Kovalev et al., 2015; Livanova et al., 2016) host populations of *I. persulcatus*, with recent evidence of their expansion toward central and western parts of Fennoscandia (Laaksonen et al., 2017; Pakanen et al., 2020; Tokarevich et al., 2011). Both tick species rely on host animals regarding the expansion of their geographical distribution. Birds, for example, are among the most important dispersal hosts for both species (Buczek et al., 2020; Hornok et al., 2020; Toma et al., 2021). We would expect a patchier distribution of *I. persulcatus* at its southernmost distribution limit should the species be vectorized mainly by animals with high mobility (Shigesada et al., 1995). Furthermore, major southwards and westwards movements of migratory birds occur in late summer/autumn in northern Europe. As the Finnish *I. persulcatus* populations (and their source populations) are no longer active during this period, it is likely that they are seldom transported south or southwest by migrating birds. During the northward migration in spring, there are few places from where birds could acquire *I. persulcatus*—mostly inland areas in Estonia (Katargina et al., 2015) and Latvia (Capligina et al., 2020). Correspondingly, no *I. persulcatus* were detected in a recent survey of ticks parasitizing migrating birds in Finland (Sormunen et al., 2022). Hence, the timing and shortness of the activity period of *I. persulcatus* appears to contribute to limiting their distributional range in Finland, the mechanism probably being linked to the mobility of host animals. The sharp southernmost distribution limit of *I. persulcatus* in Finland could be due to the diffusive range expansion of the species (e.g., Shigesada et al., 1995) where ticks are transported with hosts with low mobility, such as voles (Norrdahl & Korpimäki, 1995).

On the other hand, hybridization, or competition through hybridization, i.e., converting a cumulatively increasing portion of the population of competing species into hybrids (Todesco et al., 2016) could explain the absence of *I. persulcatus* from southern Finland. Hybrids formed by male *I. persulcatus* mating with female *I. ricinus* occur more commonly than vice versa (Kovalev et al., 2016). Such higher affinity toward hybrid formation for one species may lead to the decline of the other species in areas of species co‐occurrence. As southern Finland hosts abundant populations of *I. ricinus* (Sormunen, Andersson, et al., 2020), it appears possible that a small number of arriving *I. persulcatus* could be hybridized to local extinction. However, screening of approximately 3800 of the crowdsourced tick samples for *I. ricinus* × *I. persulcatus* hybrids using a duplex qPCR assay identified only 35 such hybrids (Laaksonen et al., 2018). Hence, hybridization appears to have a minor influence on our results.

The results of this study were obtained by analyzing crowdsourced data on the presence of *I. ricinus* and *I. persulcatus* in Finland. As our data were collected by citizens, the number of observations is intrinsically correlated with the number of observers (Cretois et al., 2021). This sampling bias may—in part—explain why the observations of both species were related to the occurrence of the urban area and the number of inhabitants. Together with differences in activity patterns between the two ixodids, this sampling bias may partly explain the interspecific differences in environmental associations observed in this study (Welvaert & Caley, 2016). More specifically, the longer activity period of *I. ricinus* compared with *I. persulcatus* leads to a higher probability to encounter *I. ricinus* than *I. persulcatus*. This difference may transmit to the number of observations that citizens make of each species. The effect may be further amplified due to the concurrency of the second activity peak of *I. ricinus* and the main summer holiday season in Finland (Pääkkönen & Hanifi, 2011; Sormunen, Kulha, et al., 2020). Despite this, the two species were observed in similar quantities from their sympatric area of occurrence (*N* = 1232, 1078 for *I. ricinus* and *I. persulcatus*, respectively). Still, the imbalance of species observations in our data may influence the results. Moreover, uncertainties in, e.g., geographic information provided along the tick samples may have introduced bias in the results despite our efforts to account for the bias in the quantification of environmental variables.

We opted not to use the information on host density as it was only available for a small subset of possible hosts (over 200 reported hosts for both species; Bowmann & Nuttall, 2008; Uspensky, 2016), observations covered the study region only partially, and/or the spatial resolution of the data was too coarse. Moreover, none of the potential hosts of either species is known to have a distribution pattern that matches with the tick observations in our data. As such, we regard data on host density/occurrence as suboptimal for analyzing the niche differences of ectoparasites dependent on host animals for transportation and which may use various hosts for which occurrence data are not available. Furthermore, ecologically credible explanations for the exclusion of *I. persulcatus* from urban areas and rocky and elevated habitats (or vice versa for *I. ricinus* and moraine) based on host animal densities/presence are missing. Should the observed differences be due to factors associated with host animals, they are more likely to be linked to the activity patterns of ticks and movement patterns of host animals during the growing season (for which data are even more sparse) than the densities of host species. Particularly, given the differences in activity patterns between the two ixodids, there is a chance that the tick species encounter different hosts and/or are transported by them differently. Thus, differences in host animal movement patterns and activity may influence the different distributions of the two ixodids in Finland.

## 5 CONCLUSIONS

Our observations suggest that in the area of species co‐occurrence, *I. ricinus* and *I. persulcatus* may colonize different niches. However, despite that we discovered a statistically significant difference in environmental associations between the two species, the differences are difficult to explain based on ecological discrepancies between the species. Hence, it is likely that distribution history rather than environmental factors explains the observed broad‐scale distribution patterns of *I. ricinus* and *I. persulcatus* in the area of species co‐occurrence in Finland.

The sharp southernmost distribution limit of *I. persulcatus* in Finland, visible in the crowdsourced occurrence data, was not explained by the environmental factors considered in this study. Hence, we argue that the recent establishment and ongoing dispersion of *I. persulcatus* in Fennoscandia rather than environmental conditions explain the current absence of *I. persulcatus* from southernmost Finland.

## AUTHOR CONTRIBUTIONS

**Tero Klemola:** Conceptualization (equal); funding acquisition (lead); investigation (supporting); project administration (lead); supervision (lead); writing – review and editing (supporting). **Niko Kulha:** Conceptualization (equal); data curation (lead); formal analysis (lead); funding acquisition (supporting); investigation (lead); methodology (lead); resources (supporting); software (lead); validation (lead); visualization (lead); writing – original draft (lead); writing – review and editing (lead). **Maija Lamppu:** Data curation (equal); writing – review and editing (supporting). **Kalle Ruokolainen:** Conceptualization (supporting); formal analysis (supporting); investigation (supporting); methodology (equal); supervision (equal); validation (supporting); visualization (supporting); writing – review and editing (supporting). **Jani J. Sormunen:** Conceptualization (equal); data curation (equal); funding acquisition (lead); investigation (equal); methodology (supporting); project administration (equal); supervision (equal); validation (equal); visualization (supporting); writing – original draft (supporting); writing – review and editing (equal). **Eero J. Vesterinen:** Conceptualization (supporting); funding acquisition (lead); project administration (equal); supervision (lead); writing – review and editing (supporting).

## CONFLICT OF INTEREST

The authors declare that the research was conducted in the absence of any commercial, financial, or other relationships that could be construed as a potential conflict of interest.

## Data Availability

The tick data and R‐code used for analysis are available at: https://doi.org/10.6084/m9.figshare.21594207.v1. The used environmental variables were openly available under the license CC‐BY‐4.0.

## Appendix

**TABLE A1:** Environmental variables used to examine niche differentiation of *Ixodes ricinus* and *Ixodes persulcatus* in Finland. Prior to use, the sea cover data were clipped with land mask derived from National Land Survey Finland. The climatic variables are averages from years 1980 to 2010. Ferry routes were removed from the national road database (Väylävirasto, 2018).

Variable group	Variable name	Data source	Original format	Minimum mapping unit	Calculated statistics	Citation
Soil variables	Bedrock and boulders	Corine Land Cover 2012 / EU	Polygon	25 ha	Proportional coverage (%)	European Union, Copernicus Land Monitoring Service (2012)
	Coarse‐grained soil	Corine Land Cover 2012 / EU	Polygon	25 ha	Proportional coverage (%)	European Union, Copernicus Land Monitoring Service (2012)
	Unsorted glacial deposits	Corine Land Cover 2012 / EU	Polygon	25 ha	Proportional coverage (%)	European Union, Copernicus Land Monitoring Service (2012)
	Fine‐grained soil	Corine Land Cover 2012 / EU	Polygon	25 ha	Proportional coverage (%)	European Union, Copernicus Land Monitoring Service (2012)
	Sludge	Corine Land Cover 2012 / EU	Polygon	25 ha	Proportional coverage (%)	European Union, Copernicus Land Monitoring Service (2012)
	Clay	Corine Land Cover 2012 / EU	Polygon	25 ha	Proportional coverage (%)	European Union, Copernicus Land Monitoring Service (2012)
	Organic soil	Corine Land Cover 2012 / EU	Polygon	25 ha	Proportional coverage (%)	European Union, Copernicus Land Monitoring Service (2012)
Surface water cover variables	Lake	Finnish Environment Institute	Polygon	Lakes and reservoirs >200 m^2^	Proportional coverage (%)	Järvi 10 / Finnish Environment Institute (SYKE)
	Sea	Finnish Environment Institute	Polygon	1 / 50,000	Proportional coverage (%)	Meri 10 / Finnish Environment Institute (SYKE)
	Surface water (lake + sea)	Finnish Environment Institute	Polygon	Combination of lake and sea layers	Proportional coverage (%)	Järvi 10 & Meri 10 / Finnish Environment Institute (SYKE)
	River	Finnish Environment Institute	Line	All river segments with catchment area > 10 km^2^	Length	Uomaverkosto / Finnish Environment Institute (SYKE)
Climatic variables	Growing season length	Finnish Meteorological Institute	Raster	10 km^2^	Mean	Aalto et al. (2016)
	Temperature sum of growing season	Finnish Meteorological Institute	Raster	10 km^2^	Mean	Aalto et al. (2016)
	Precipitation sum of growing season	Finnish Meteorological Institute	Raster	10 km^2^	Mean	Aalto et al. (2016)
	Mean temperature	Finnish Meteorological Institute	Raster	1 km^2^	Mean	Aalto et al. (2016)
	Snow season length	Finnish Meteorological Institute	Raster	10 km^2^	Mean	Aalto et al. (2016)
Vegetation variables	Tree canopy cover	Natural Resources Institute Finland	Raster	256 m^2^	Mean	Mäkisara et al. (2019)
	Proportion of deciduous trees	Natural Resources Institute Finland	Raster	256 m^2^	Proportional coverage (%)	Mäkisara et al. (2019)
	Mean tree age	Natural Resources Institute Finland	Raster	256 m^2^	Mean	Mäkisara et al. (2019)
	Standard deviation of mean tree age	Natural Resources Institute Finland	Raster	256 m^2^	Mean	Mäkisara et al. (2019)
	Proportion of young forest (< 30 years of age)	Natural Resources Institute Finland	Raster	256 m^2^	Proportional coverage (%)	Mäkisara et al. (2019)
Human variables	Number of inhabitants (in 2015)	Statistics Finland	Polygon	1 km^2^	Sum	Statistics Finland (2020)
	Road length	Finnish Transport Infrastructure Agency	Line	1 / 10,000	Sum	Finnish Transport Infrastructure Agency (2018)
	Summer cottages	Digital and Population Data Services Agency Finland	Point	All buildings with known coordinates and address	Count	Digital and Population Data Services Agency (2016)
Topographic variables	Elevation	National Land Survey Finland	Raster	100 m^2^	Mean	National Land Survey of Finland (2019)
Land cover variables	Urban area	Corine Land Cover 2012 / EU	Polygon	25 ha	Proportional coverage (%)	European Union, Copernicus Land Monitoring Service (2012)
	Arable land	Corine Land Cover 2012 / EU	Polygon	25 ha	Proportional coverage (%)	European Union, Copernicus Land Monitoring Service (2012)
	Peatland	Corine Land Cover 2012 / EU	Polygon	25 ha	Proportional coverage (%)	European Union, Copernicus Land Monitoring Service (2012)

**TABLE A2:** Pearson's *r* between the five variables with the estimated *p*‐value <5%.

	Elevation	Number of inhabitants	Bedrock and boulders	Unsorted glacial deposits	Road length
Elevation	—	—	—	—	—
Number of inhabitants	0.03	—	—	—	—
Bedrock and boulders	0.37	0.05	—	—	—
Unsorted glacial deposits	0.07	−0.17	−0.20	—	—
Road length	−0.05	0.77	−0.04	−0.11	—

**TABLE A3:** Results of the PCA performed using with each environmental variable considered in this study.

Rank of environmental dimension	Eigenvalue	Cumulative variance percent
1	4.61	16.48
2	3.46	28.84
3	3.24	40.43
4	2.74	50.21
5	1.86	56.87
6	1.37	61.76
7	1.33	66.49
8	1.03	70.18
9	0.99	73.72
10	0.97	77.17
11	0.88	80.30
12	0.83	83.28
13	0.75	85.96
14	0.67	88.34
15	0.64	90.62
16	0.54	92.55
17	0.51	94.38
18	0.46	96.01
19	0.30	97.08
20	0.25	97.96
21	0.18	98.60
22	0.15	99.13
23	0.08	99.40
24	0.07	99.64
25	0.05	99.83
26	0.03	99.95
27	0.01	99.99
28	0.00	100.00
